# Mapping and assessment of personal and professional development skills in a pharmacy curriculum

**DOI:** 10.1186/s12909-016-0533-4

**Published:** 2016-01-15

**Authors:** Elsy Ramia, Pascale Salameh, Imad F. Btaiche, Aline Hanna Saad

**Affiliations:** Department of Pharmacy Practice, School of Pharmacy, Lebanese American University, P.O.Box: 36 (S23), Byblos, Lebanon; School of Pharmacy, Lebanese American University, P.O.Box: 36, Byblos, Lebanon

**Keywords:** Mapping, Curriculum, Personal and professional development, Self-assessment, Leadership, Innovation and entrepreneurship, Professionalism

## Abstract

**Background:**

Health sciences programs are increasingly expanding their curricula to bridge foundational scientific knowledge with needed skills to practice and patient care. The primary objectives of this study are to 1) assess whether the personal and professional development (PPD) subdomains (self-assessment, leadership, innovation and entrepreneurship, and professionalism) are integrated in a pharmacy curriculum; and 2) identify any gaps related to the subdomains’ learning objectives.

**Methods:**

Four different mapping activities were completed to create a comprehensive mapping plan regarding the integration of the PPD subdomains in the curriculum. The first mapping activity entailed matching the school’s program educational outcomes (PEOs) to these subdomains (Step 1). Mapping of the enacted curriculum by faculty (Step 2) and learned curriculum by students (Step 3) were also completed in order to evaluate the integration of these subdomains in the curriculum. Finally, Step 4 involved mapping of the assessed curriculum by analyzing the progress of students on PPD-related competencies using standardized scoring rubrics and the correlation between students’ and facultys' assessments with regard to matching competencies. The Cochrane’s Q test and the Cohen’s kappa coefficient were used in the statistical analysis of parametric data.

**Results:**

The subdomains were found to be woven across curricular, co-curricular, and extra-curricular activities based on the four different mapping activities. Faculty and students agreed that the PPD competencies are integrated in the curriculum; provided example courses, experiences and activities; and identified areas of further improvements. The completed mapping activities drove the development of action plans for remediation of identified gaps in the curriculum.

**Conclusion:**

Mapping activities showed the sequential integration of the PPD skills at different depths and breadths in the curriculum. This study provides an example to health sciences schools on the incorporation of the PPD skills in their curricular, co-curricular and extra-curricular activities as current accreditation standards have directed Pharmacy programs to integrate and enforce them in their curricula.

## Background

In a constantly evolving healthcare environment, preparing students for a lifetime of change and emergent opportunities is a challenging responsibility in higher education. Pharmacy students must acquire needed knowledge, and couple it with personal and professional development (PPD) skills, to allow for the development of well-rounded health care professionals capable of providing holistic patient care. These PPD skills involve setting clear goals and action plans, identifying strengths and areas for improvement and reflecting on personal and professional progress [1]. They ultimately equip the students with the needed tools to become lifelong learners. The Center for the Advancement of Pharmacy Education (CAPE) 2013, in its fourth version of educational outcomes, expanded its domains beyond knowledge and skills to include the affective domain (Domain 4), reflecting the importance of personal attributes and professional skills to the practice of pharmacy. The CAPE outcomes were built around 4 broad domains to guide the education of pharmacists in 1) foundational knowledge, 2) essentials for practicing pharmacy and delivering patient-centered care, 3) effective approaches to practice and care, and 4) the ability to develop personally and professionally. This expansion emphasizes the necessity to develop students personally and professionally with a mindset of four skills, namely self-awareness, leadership, innovation and entrepreneurship, and professionalism [2].

Hawthorn and Watts defined self-awareness as “awareness of the distinctive characteristics (abilities, skills, values and interests) that define the kind of person one is, and the kind of person one wishes to become” [3]. Similarly, a self-aware individual is one able to “examine and reflect on personal knowledge, skills, abilities, beliefs, biases, motivation, and emotions that could enhance or limit personal and professional growth” [2]. This ability to accurately assess one’s weaknesses and strengths is vital to students and practitioners as it generates a capacity for finding an effective balance in daily practice, setting learning goals, and reflecting on accomplishments. It generates a balance of confidence and caution, of persistence and flexibility, of knowing what to tackle and what to abandon, and a balance of self-reward without self-delusion [4].

A leader is entrusted to “demonstrate responsibility for creating and achieving shared goals, regardless of position” [2]. Developing leadership skills has never been more important for the pharmacy profession than it is today, for future pharmacists must be prepared as citizen leaders to execute the promised vision of pharmacy [5]. In its statement on leadership, the American Society of Health-System Pharmacists (ASHP) emphasizes that leadership is a “professional obligation” for all pharmacists, where it calls every pharmacist to function as a “leader in the safe and effective use of medications” [6]. Lifelong learning and leadership should hence be included as objectives in pharmacy educational programs. Students and practitioners must be willing to learn and lead as they strive towards professional excellence, and assume a leadership role in ensuring patients’ understanding and involvement in their care [7]. Accordingly, the Accreditation Council for Pharmacy Education (ACPE) Standards 2007 and 2016 include learning outcomes of leadership for professional pharmacy programs, specifically mentioned for the program mission and goals, school governance, students’ admission criteria, representation, and professional behavior [5].

Innovation is defined as engaging in “innovative activities by using creative thinking to envision better ways of accomplishing professional goals” [2]. Entrepreneurial spirit, in its intended positive connotations, includes additional elements such as uniqueness, adaptability, developing potential and creating new opportunities. These skills now constitute a necessity for future graduates, as they are entrusted to envision the dynamic future of pharmacy practice. Accordingly, pharmacy curricula are responsible to stimulate pharmacy students’ creativity and provide them with curricular and extracurricular opportunities to develop and nurture their entrepreneurial abilities [7].

Compared to the medical curricula that emphasize the values and attributes of professionalism, Pharmacy has given more importance to its behavioral aspects and their application in practice such as initiative, empathy, lifelong learning, and responsibility to name few [8]. Hence, a professional pharmacy student is expected to “exhibit behaviors and values that are consistent with the trust given to the profession by patients, other healthcare providers, and society” [2]. As professionalism is mainly comprised of attitudes and behaviors, published literature on the subject reflects that it may not be merely taught, but would rather be swayed through hidden curricula, and through a set of experiences that shape students skills and abilities in the right direction [8, 9]. Hidden curricula comprise a set of values, attitudes and behaviors that are not formally taught to students, but influenced by the learning environment and role modeling [8].

Mapping is a valid and reliable assessment mechanism of charting various elements of the curriculum that offers insight to programs’ strengths and deficiencies, and provides opportunities to improve the quality of pharmacy education [10]. It is a continuous quality assurance strategy that is mandated by accreditors, such as ACPE. Through mapping, faculty gain an understanding of their programs regarding course content, delivery, depth and breadth of covered competencies. The literature usually reports mapping completed by faculty (enacted curriculum) and focuses on knowledge acquired through basic sciences. Curricula can be appraised from different standpoints including the enacted curriculum (what faculty do in the classroom as per syllabi), the learned curriculum (what students experience and learn), and the assessed curriculum (for which we actually test or assess competencies) [11]. As such, the enacted curriculum reflects on what faculty teach; the enacted curriculum on what students experience and think they have learned; and the assessed curriculum on students ‘actual achievement of competencies.

The primary objectives of this study are to 1) assess whether self-assessment, leadership, innovation and entrepreneurship, and professionalism learning objectives are integrated in a pharmacy curriculum through the mapping of the taught/enacted, learned, and assessed curricula, and 2) identify any gaps related to these learning objectives in an effort to improve the curriculum. Considering the scarcity of literature related to this subject, this study will provide schools and colleges of pharmacy and health sciences with a prototype for the assessment of integrating PPD skills in curricular, extra-curricular and co-curricular activities.

## Methods

### Tool

The CAPE Educational Outcomes 2013, Domain 4 on PPD skills, were utilized as a template to map the achievement of self-assessment, leadership, innovation and entrepreneurship, and professionalism learning objectives in the pharmacy curriculum of the professional degree program at the Lebanese American University (LAU) School of Pharmacy (SOP).

**Fig. 1:**
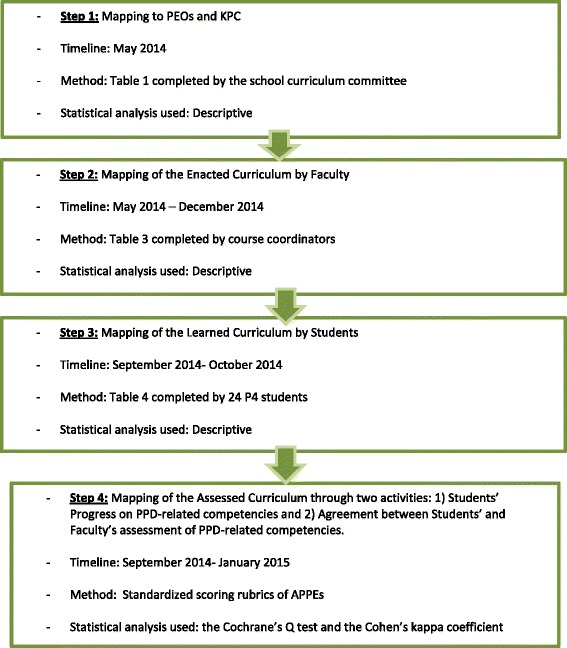
The four mapping steps

Four different mapping activities were completed to create a comprehensive mapping plan as depicted in Fig. 1:

The first mapping activity (Step 1) entailed matching the school’s program educational outcomes (PEOs) and corresponding key performance criteria (KPC) to the four subdomains of self-awareness, leadership, innovation and professionalism of Domain 4, CAPE 2013. Next, all courses delivered across the four professional years were screened and those found to cover at least one of the four subdomains were identified. The enacted curriculum was then mapped with respective course coordinators (Step 2). Course coordinators were provided with the reasons for mapping and clarification of the evidence needed to meet the competencies (examples of selective evidence included course learning objectives, assignments, evaluation forms, and scoring rubrics). Moreover, students’ co-curricular (orientation) and extra-curricular (workshops; societies and clubs) activities were mapped to the aforementioned subdomains. This mapping activity extended from May 2014 to December 2014.

Similarly, 32 fourth professional year (P4) students were invited to map the learned curriculum by providing anonymous feedback on the PPD subdomains (Step 3). The concept, value and metrics (meet/don’t meet) used for the mapping were explained to the students. Students were asked to 1) reflect on their learning experiences and decide if the competencies in question have or have not been met throughout the curriculum; 2) provide examples of courses or activities where the competencies are met; and 3) offer suggestions for improvements if necessary.

The last phase of the study involved the assessed curriculum (Step 4). The standardized scoring rubric of Advanced Pharmacy Practice Experiences (APPEs) was mapped to the selected subdomains in order to verify that they are assessed through the APPE curriculum. The scoring rubric, that uses a scale of 1 to 5 (1-does not know, 2-knows, 3-knows how, 4-shows how, and 5-does), allows the students to complete self-assessment at baseline, midpoint and end of rotations, and also enables the faculty to assess students at midpoint and end of rotation. Twenty-two competencies are assessed in this scoring rubric focusing mostly on core performance areas, with eight of them addressing the PPD skills. The progress of students on PPD-related competencies was assessed for 32 fourth professional year pharmacy students (P4) as they completed 5 consecutive APPEs spanning from September 2014 to January 2015. Since each APPE is completed over 4 weeks, students’ progress was accordingly followed for 20 weeks (5 months). Agreement between students’ and faculty’s assessments with regard to PPD-related competencies using the same standardized APPE scoring rubric over a period of 5 months (20 weeks) was also analyzed. Regarding the latter activity of Step 4, it is to be noted that a preceptor could have rated more than one student.

The study protocol was approved as “exempt” by the Lebanese American University (LAU) Institutional Review Board (IRB). No formal consents were required as the data was pulled from the school's curriculum committee database. No identifiers were used and data was processed while maintaining complete confidentiality and anonymity.

### Statistical analysis

This is a descriptive, cross-sectional study based on students’ learning outcomes and curricular data sets. Data from Steps 1 through 3 were analyzed using descriptive frequencies and percentages as mapping is not usually a procedure that is studied with parametric statistics. Pooled data was applied for descriptive results (Steps 1-3) while paired data was used when evaluating students’ progress and agreement between preceptors and students on PPD-related competencies (Step 4). In Step 4, statistical analysis was performed using SPSS (IBM version 18 software, 2009) and the Cochrane’s Q test and the Cohen’s kappa coefficient were used. The Cochrane’s Q test was used to compare the proportion of students who had a mean assessment level of 4 or more between baseline, midpoint and end of rotation. The Cohen’s kappa coefficient was used to assess agreement between students and preceptors at midpoint and end of rotation regarding the achievement of the PPD-related competencies. A p value below 0.05 was considered to be statistically significant.

## Results

The four PPD subdomains, including self-awareness, leadership, innovation and entrepreneurship, and professionalism, were found to be woven throughout the curriculum across curricular (courses from all four professional years), co-curricular (orientation sessions, workshops and seminars), and extra-curricular activities (organizations). Mapping findings also showed the sequential integration of these subdomains at different depths and breadths in the curriculum.

### Step 1- Mapping to PEOs and KPC

The mapping results showed that the four subdomains are addressed in the PEOs. The subdomains matched three out of the 14 PEOs namely PEOs 12, 13 and 14 with corresponding KPC 12.A, 12.D, 13.A, 13.C, 14.B, 14.C, 14.D, and 14.E (Table 1). PEO 14 addresses self-assessment, leadership and innovation by having the student identify his/her learning preferences and describe his/her strengths and self-limitations, develop strategies for overcoming weaknesses, manage time appropriately and efficiently, and display confidence and self-motivation. PEOs 12 and 13 address leadership and professionalism as the student develops a sense of pride, dignity and purpose concerning pharmacy, demonstrates communications that would be helpful when there are ethical conflicts, identifies local and international regulations affecting pharmacy practice, and demonstrates the ability to counsel patients while guaranteeing the confidentiality of their prescriptions and medical records. Innovation and entrepreneurship were however identified as areas of needed improvement, although addressed under PEO 14, as initiative and innovative applications were not explicitly included. This was communicated to the school’s Assessment and Evaluation Committee for further evaluation and to the Curriculum Committee for follow-up on the findings.

**Table 1:** Mapping of the personal and professional development skills to PEOs and KPC

Subdomains of Personal and Professional Development	Program Educational Outcomes (PEOs)	Key Performance Criteria (KPC)
Sub-domain 1: Self-awareness Sub-domain 2: Leadership Sub-domain 3: Innovation and entrepreneurship	PEO 14: Exhibit intellectual curiosity and personal commitment to ensure ongoing professional competency by identifying and analyzing emerging issues, products, and services that may impact patient-specific and population- based pharmaceutical care.	KPC 14.B: Identify her/his learning preferences and describe their strengths and self-limitations.
		KPC 14.C: Develop strategies for overcoming the weaknesses.
		KPC 14.D: Manage time appropriately and efficiently.
		KPC 14.E: Display confidence and self-motivation.
Sub-domain 2: Leadership Sub-domain 4: Professionalism	PEO 12: Explain the moral standards and professional conduct and discuss the ethical obligations related to the profession of pharmacy in order to resolve ethical conflicts and dilemmas.	KPC 12.A: Develop a sense of pride, dignity and purpose concerning pharmacy.
		KPC 12.C: Demonstrate communications that would be helpful when there are ethical conflicts.
	PEO 13: Demonstrate compliance with local, state, federal and international regulations affecting pharmacy practice.	KPC 13.A: Identify local and international regulations affecting pharmacy practice (accreditation, education, licensures…).
		KPC 13.D: Demonstrate ability to counsel patients while guaranteeing the confidentiality of their prescriptions and medical records.

### Step 2- Mapping of the enacted curriculum by faculty

The PPD skills are introduced to students in the first professional year (P1) through various didactic and experiential courses including Professional Communication (PHA 322), Pharmacy Practice and Ethics (PHA 325), Pharmacy Management (PHA 333), and Pharmacy Practice Management I (PHA 398). In the second professional year (P2), leadership and professionalism are reinforced in the Dispensing and Pharmaceutical Care course (PHA 449) and the Introductory Pharmacy Practice Experiences (IPPEs- PHA 497 and 499). These skills are further developed during the third and fourth professional years (P3 and P4) through more IPPEs and APPEs. Table 2 details the didactic and experiential courses that were mapped from the various professional years and Table 3 presents the enacted curriculum mapping results. Important findings from this mapping included the following: 1) some confusion was noted among faculty between leadership and management delivery in the curriculum; 2) there is a need to enrich the pharmacy management content of the curriculum with application of the theoretical concepts delivered; 3) outreach clinical experiences are all part of elective APPEs that expose P4 students to cultural competence, and provide them with leadership and innovative opportunities to deliver care to underserved populations at these clinics; 4) not all students organizations have clearly developed and documented learning objectives; and 5) the career opportunities course that incorporated all of the subdomains in its objectives is an elective course that is not regularly offered.

**Table 2:** Mapped courses

Professional Year	Courses Mapped	PHA Number	Depth of Delivery
Professional Year 1 (P1)	Professional Communication	PHA 322	Apply
	Pharmacy Practice and Ethics	PHA 325	Introduce
	Pharmacy Management	PHA 333	Introduce
	Pharmacy Practice Management I	PHA 398	Reinforce/Apply
Professional Year 2 (P2)	Dispensing and Pharmaceutical Care	PHA 449	Apply
	Introductory Pharmacy Practice Experiences-IPPE	PHA 497	Apply
	Pharmacy Practice Management II	PHA 499	Apply
Professional Year 3 (P3)	Seminar	PHA 515	Apply
	Professional Pharmacy Practice – Hospital/Drug Information Center Experience	PHA 570	Apply
	Professional Pharmacy Practice –Community Experience	PHA 571	Apply
	Professional Pharmacy Practice- Inpatient Care Experience	PHA 572	Apply
Professional Year 4 (P4)	Required Advanced Pharmacy Practice Experiences- APPEs	PHA 670-671-672-673	Apply
	Elective Advanced Pharmacy Practice Experiences- APPEs	PHA 650 (17 different elective experiences are available to students)	Apply

**Table 3:** Mapping of the enacted curriculum

PPD Skills	Example Learning Objectives per the CAPE Educational Outcomes 2013- Domain 4	Example Courses (Course Number)/ Co and Extra-curricular Activities	Select Evidence for Achievement of Learning Objectives based on Students and Curricular Data Sets
Self-awareness	4.1.1 Use of self-awareness to regulate one's own thinking and learning	• IPPEs and APPEs • PharmD mentoring • Student advising • P4 workshop on emotional intelligence • P4 workshop on interviewing skills	• Baseline/Midpoint/Final Point IPPEs and APPEs Scoring Rubrics (competency based assessment in IPPEs and APPEs) • Day to day interaction and feedback provided to students • Twice yearly mentor-mentee meetings using a mentoring plan • Regular meetings with advisors to track performance
	4.1.2 Maintain motivation, attention, and interest during learning and work-related activities		
	4.1.3 Identify, create, implement, evaluate, and modify plans for personal and professional development for the purpose of individual growth.		
	4.1.4 Approach tasks with a desire to learn		
	4.1.5 Demonstrate persistence and flexibility in all situations; engaging in help seeking behavior when appropriate.		
	4.1.6 Strive for accuracy and precision by displaying a willingness to recognize, correct, and learn from errors.		
	4.1.7 Use constructive coping strategies to manage stress		
	4.1.8 Seek personal, professional, or academic support to address personal limitations.		
	4.1.9 Display positive self-esteem and confidence when working with others.		
Leadership	4.2.1. Identify characteristics that reflect leadership versus management.	• PHA 567 - Career Opportunities • Students Societies: 3 societies available at the school • Three Voluntary Outreach Clinics • Advanced Pharmacy Practice Experiences- required and elective APPEs: PHA 670 - 671 - 672 - 673 - and 650 s • Workshops	• Documented students learning objectives (SLO) in syllabi of identified courses • The mission of NAPHASS-one student society- is to cultivate leadership in pharmacy students and create a public image of pharmacists, in the Lebanese communities, as patient care providers and medication use experts. • Example of Students Learning Objectives from the outreach activities: - Provide students with the opportunity to practice medicine, nursing and pharmacy in a challenging environment where innovation and the social determinants matter. • SLOs in the APPEs syllabi • PharmD students manual • Effective team building workshop, provided to P4 students by the Outreach and Civic Engagement (OCE) Unit at LAU
	4.2.2. Identify the history (e.g., successes and challenges) of a team before implementing changes.		
	4.2.3. Develop relationships, value diverse opinions, and understand individual strengths and weaknesses to promote teamwork.		
	4.2.4. Persuasively communicate goals to the team to help build consensus.		
	4.2.5. Empower team members by actively listening, gathering input or feedback, and fostering collaboration.		
Innovation and Entrepreneurship	4.3.1. Demonstrate initiative when confronted with challenges.	• PHA 333 - Pharmacy Management	• Example of SLOs documented in the syllabus: - Evaluate the skills needed for success - Appraise readiness to become an entrepreneur - Describe the ability to mesh personal and business goals - Describe the ability to conceptualize and plan - Explain the personal, financial and marketing considerations
	4.3.2. Develop new ideas and approaches to improve quality or overcome barriers to advance the profession.		
	4.3.3. Demonstrate creative decision making when confronted with novel problems or challenges.		
	4.3.4. Assess personal strengths and weaknesses in entrepreneurial skills.		
	4.3.5. Apply entrepreneurial skills within a simulated entrepreneurial activity.		
	4.3.6. Conduct a risk-benefit analysis for implementation of an innovative idea or simulated entrepreneurial activity.		
Professionalism	4.4.1. Demonstrate altruism, integrity, trustworthiness, flexibility, and respect in all interactions.	• PHA 325 - Pharmacy Practice and Ethics • PHA 322 - Professional Communication • PHA 449 - Dispensing and Pharmaceutical Care • PHA 515- Seminar • IPPEs and APPEs • Students Societies - Orientation Sessions -Service on School Committees • Workshop for P4 students, given by the Outreach and Civic Engagement Unit, entitled: “Hands on Strategies for Building Confident and Collaborative Star Performers”	• Example of SLOs documents in syllabi of identified courses • IPPEs and APPEs scoring rubrics • Students societies activities • Orientation Sessions for IPPEs and APPEs- Checklist • PharmD student manual • Documentation of students service on school committees

### Step 3- Mapping of the learned curriculum by students

Twenty four out of 32 (75 %) P4 students completed the mapping activity (Table 4). On average, 92 % and 85 % of students agreed that the self-awareness and leadership subdomains and their corresponding learning objectives are met in the curriculum, respectively. Similar to facultys' input in the enacted curriculum mapping, students agreed to the need of clarifying the characteristics that reflect leadership versus management. Students also reported deficiencies in opportunities for innovation and entrepreneurship in the curriculum, with an average of 64 % agreeing that this is met in the curriculum. As for the professionalism subdomain, 99 % of students agreed that it is being thoroughly addressed. Students provided insightful suggestions for improvement, namely 1) needed workshops on coping with stress; 2) providing practical, rather than theoretical, opportunities for leadership; and 3) enriching the curriculum with entrepreneurial experiences that would develop creative decision making skills for students.

**Table 4:** Mapping of the learned curriculum by 24 P4 students

PPD Skills	Example Learning Objectives per the CAPE Educational Outcomes 2013- Domain 4	Overall % Meet	Average Overall % Meet	Examples from the Curriculum	Suggestions for Improvement
Self-awareness	4.1.1 Use of self-awareness to regulate one's own thinking and learning	92	92	• Advising • Orientations • IPPEs and APPEs • Mentorship • P4 admission interviews • IPPEs and APPEs scoring rubrics • Meeting with preceptors • Self- assessment after oral presentations • Emotional Intelligence workshop	• Motivate students to develop self-assessment skills and other personal/ professional growth skills. • Include more workshops in the program starting P1 year and similar to the one offered to P4 students. • Help students to better cope with stress.
	4.1.2 Maintain motivation, attention, and interest during learning and work-related activities	96			
	4.1.3 Identify, create, implement, evaluate, and modify plans for personal and professional development for the purpose of individual growth.	96			
	4.1.4 Approach tasks with a desire to learn	92			
	4.1.5 Demonstrate persistence and flexibility in all situations; engaging in help seeking behavior when appropriate.	96			
	4.1.6 Strive for accuracy and precision by displaying a willingness to recognize, correct, and learn from errors.	96			
	4.1.7 Use constructive coping strategies to manage stress	84			
	4.1.8 Seek personal, professional, or academic support to address personal limitations.	80			
	4.1.9 Display positive self-esteem and confidence when working with others.	96			
Leadership	4.2.1. Identify characteristics that reflect leadership versus management.	71	85	• Mentorship • Advising • Professional Communications-(PHA 322) • Seminar -(PHA 515) • Ethics and pharmacy practice- (PHA 325) • IPPEs and APPEs • Students involvement in school committees • Conflict Management and Emotional Intelligence Workshops • Leadership workshop • Inter-professional education experiences	• More Workshops • The curriculum provides more theoretical than practical opportunities to develop leadership skills and lead the profession of pharmacy.
	4.2.2. Identify the history (e.g., successes and challenges) of a team before implementing changes.	88			
	4.2.3. Develop relationships, value diverse opinions, and understand individual strengths and weaknesses to promote teamwork.	84			
	4.2.4. Persuasively communicate goals to the team to help build consensus.	92			
	4.2.5. Empower team members by actively listening, gathering input or feedback, and fostering collaboration.	92			
Innovation and Entrepreneurship	4.3.1. Demonstrate initiative when confronted with challenges.	67	64	• IPPES • Seminar – (PHA 515) • Elective APPE: Regulatory affairs • Elective APPE: Primary care • Elective APPE: Medication safety • Elective APPE: Industrial rotation • Students organizations	• More attention should be given to this point: as students, we sometimes tend to follow instructions and plans and forget to come up with new ideas. • Students should give more input on how to apply their knowledge for the benefit of others. • Need more creative ways to deliver the objectives of courses by ensuring students engagement in application activities. • Need more business/management content in the curriculum.
	4.3.2. Develop new ideas and approaches to improve quality or overcome barriers to advance the profession.	63			
	4.3.3. Demonstrate creative decision making when confronted with novel problems or challenges.	59			
	4.3.4. Assess personal strengths and weaknesses in entrepreneurial skills.	71			
	4.3.5. Apply entrepreneurial skills within a simulated entrepreneurial activity.	55			
	4.3.6. Conduct a risk-benefit analysis for implementation of an innovative idea or simulated entrepreneurial activity.	67			
Professionalism	4.4.1. Demonstrate altruism, integrity, trustworthiness, flexibility, and respect in all interactions.	96	96	• Workshops • Orientation sessions at the beginning of APPEs and IPPEs • Professional Communications (PHA 322) • IPPES and APPES • Student organizations	• Recommend more flexibility in the program while maintaining professionalism and abiding by rules and regulations.
	4.4.2. Display preparation, initiative, and accountability consistent with a commitment to excellence.	100			
	4.4.3. Deliver patient-centered care in a manner that is legal, ethical, and compassionate.	100			
	4.4.4. Recognize that one’s professionalism is constantly evaluated by others.	100			
	4.4.5. Engage in the profession of pharmacy by demonstrating a commitment to its continual improvement.	100			

### Step 4- Mapping of the assessed curriculum

The PPD-related competencies are assessed in the curriculum as part of the standardized P4 APPEs scoring rubric and presented in Table 5. They are also assessed in the IPPEs scoring rubrics; the criteria used in the P4 students’ individual mentoring plan; and relevant course specific evaluation forms. Table 5 shows the progress of P4 students in their personal and professional skills’ development as they advance through five different required and elective APPEs over a period of 20 weeks. The correlation between students’ self-assessment and preceptors’ assessment of the PPD competencies is also detailed in Table 5. For students’ self-assessment, there was a clear and statistically significant improvement regarding all competencies as students were progressing through their rotations. A similar improvement was demonstrated through preceptors’ assessment, except for the professionalism domain related competencies where results were already close to 100 % at midpoint. Students and preceptors significantly agreed at midpoint for the majority of assessed competencies, except for professional judgment and professionalism domain where non-significant agreement was found (*p* = 0.258 and *p* = 0.855, respectively). At the end of rotations, there was full agreement about achievement of all competencies, except for one competency (22) on scientific inquiry and explanation in practice where a statistically non-significant disagreement was found.

**Table 5:** Correlation between students’ self-assessment and faculty’s assessment of the PPD skills

		Self-Assessment *N* = 32				Faculty Assessment			Midpoint agreement^c^	End of rotation agreement^c^
Subdomains	Matching Competencies^a^	Baseline	Midpoint	End of Rotation	*p*-value ^b^	Midpoint	End of Rotation	*p*-value ^b^	Kappa (*p*-value)	Kappa (*p*-value)
Self-awareness	15	15 (46.7 %)	30 (93.8 %)	32 (100 %)	<0.001	27 (84.4 %)	32 (100 %)	0.025	0.529 (0.001)	N/A^d^
	20	15 (46.7 %)	30 (93.8 %)	32 (100 %)	<0.001	24 (75.0 %)	32 (100 %)	0.005	0.333 (0.011)	N/A^d^
Leadership	16	9 (28.1 %)	27 (84.4 %)	32 (100 %)	<0.001	20 (62.5 %)	29 (90.6 %)	0.003	0.170 (0.258)	N/C^d^
	19	15 (46.7 %)	28 (87.5 %)	32 (100 %)	<0.001	20 (62.5 %)	32 (100 %)	0.001	0.385 (0.006)	N/A^d^
	21	14 (43.8 %)	27 (84.4 %)	32 (100 %)	<0.001	24 (75.0 %)	32 (100 %)	0.005	0.524 (0.002)	N/A^d^
Innovation and Entrepreneurship	16	9 (28.1 %)	27 (84.4 %)	32 (100 %)	<0.001	20 (62.5 %)	29 (90.6 %)	0.003	0.170 (0.258)	N/C^e^
	19	15 (46.7 %)	28 (87.5 %)	32 (100 %)	<0.001	20 (62.5 %)	32 (100 %)	0.001	0.385 (0.006)	N/A^d^
	22	7 (21.9 %)	22 (68.8 %)	31 (96.9 %)	<0.001	18 (56.3 %)	29 (90.6 %)	0.002	0.344 (0.044)	−0.049 (0.744)
Professionalism	13	29 (90.6 %)	32 (100 %)	32 (100 %)	0.050	31 (96.9 %)	32 (100 %)	0.317	N/C^e^	N/A^d^
	14	28 (87.5 %)	31 (96.9 %)	32 (100 %)	0.074	31 (96.9 %)	32 (100 %)	0.317	−0.032 (0.855)	N/A^d^

a: Competencies from the APPEs scoring rubric are detailed below

Competency 13- Maintain Professional-Ethical Standards

Competency 14- Demonstrates Human Relations Skills

Competency 15-Displays Conscientiousness & Follows Through/Handles Details

Competency 16- Demonstrates Professional Judgment

Competency 19- Displays Independence/Assertiveness

Competency 20- Demonstrates Personal and Professional Growth

Competency 21- Promote Team Building

Competency 22- Demonstrates Scientific Inquiry/Explanation in Practice

b: Cochrane’s Q test; c: Cohen’s kappa agreement coefficient; d: N/A = Not Applicable because of full agreement; e: N/C = Not computable

## Discussion

Health sciences education programs are increasingly emphasizing the education of the whole person to graduate life-long learners, critical thinkers, self-aware problem solvers, and empathic health care professionals [2, 4, 10, 12–14]. This study addresses the integration of the affective learning objectives regarding personal and professional skills, attitudes and attributes required for the delivery of patient-centered care. Four different mapping activities are utilized to assess these PPD skills in the curriculum. In addition, examples of curricular, co-curricular and extra-curricular activities where these learning objectives can be achieved are provided.

The study results show that self-assessment competencies are developed and evaluated within the program. Students exhibited good self-assessment skills which correlate well with faculty's assessment of their skills in the subdomains. In contrast to our findings, flawed self-assessment skills have been reported in the pharmacy and medical literature [15, 16]. Potential reasons were over-confidence, doing the assessment for the wrong reasons, as well as misaligned motivations [17]. Due to the complexity of this cognitive process, adopting a guided self-assessment strategy based on standardized and well-known criteria can help students develop a reflective thinking process, and enable them to identify areas where they lack professional expertise [17]. The literature on self-assessment in pharmacy and health science education and professional practice has been reviewed and recommendations for improvement are provided. Accordingly, self-assessment can be improved by cultivating external feedback; creating tools to improve feedback quality, including simulations and Objective Structured Clinical Examinations (OSCEs); developing a reflective thinking process that brings awareness of abilities; helping students recognize the theoretical versus the achieved value of reflection by providing benchmarks for self-assessment; and training them to maintain attentiveness and good habits of the mind [17–19].

In this study, findings related to leadership skills highlight the need to further develop theoretical (leadership vs. management) and applicable opportunities in the curriculum. Based on literature review and input from 54 institutions across the U.S., recommendations to develop and promote leadership in pharmacy students included supporting and encouraging students to accept leadership roles; establishing opportunities for leadership in student organizations; providing role models and mentors for leadership; and integration of leadership building skills throughout the curriculum, ranging from leadership electives to a leadership emphasis area for P4 students [5]. A published example involved an endeavor to promote leadership among pharmacy students through a three-credit hour elective leadership course offered to students at Regis University, Denver, Colorado. After successfully completing their hospital and community IPPEs, students participated in leadership quality presentations, selected and facilitated team building activities for pharmacy-based scenarios, created a personal mission statement, maintained a journal, facilitated leadership discussions and activities, and completed a variety of leader-development inventories to identify their strengths and opportunities for growth. Based on students’ feedback, these course assignments guided the creation of personalized leader-development tracks, and promoted lifelong learning [20].

The various mapping activities used in this study all concurred that innovation and entrepreneurship skills afford further improvement in the program. In addition to the CAPE educational outcomes 2013, two papers titled “pharmacy student entrepreneurial orientation: a measure to identify potential pharmacist entrepreneurs” and “entrepreneurial spirit in pharmacy” detail propositions for the development and improvement of innovation and entrepreneurship skills [2,21,22]. The latter suggestions include helping students assess their personal strengths and weaknesses in entrepreneurial skills; motivating students to stay intellectually curious and to maintain their entrepreneurial spirit by realizing that even the most successful individuals have also had failures and challenges in their professional journey; creating awards or honors that recognize outstanding students for their intellectual curiosity and/or an entrepreneurial spirit; providing presentation/discussion sessions with local pharmacists who have established innovative practices that meet community needs (i.e. immunizations, specialized compounding, mobile pharmacy serving homeless); helping students identify their future career aspirations, and their interest in business or pharmacy ownership, to nurture their existing entrepreneurial inclinations; and finally broadening the idea of entrepreneurship beyond the idea of ownership, to include the innovative use of resources in the marketplace, regardless of the pharmacy practice setting [2, 21–23].

Of all the PPD skills, professionalism was the first skill to be incorporated in our school curriculum. This study emphasizes the achievement of professionalism-related competencies even before students complete their APPEs. Key position papers have been published on professionalism by the ASHP and the American College of Clinical Pharmacy (ACCP) detailing its essence, traits, guiding principles and responsibilities as well as the need to incorporate it into practice [24, 25]. The papers are well incorporated into our curriculum through various orientation sessions as students are preparing for their IPPEs and APPEs. Yet, role modeling is believed to be the most important factor to improve pharmacy student professionalism during experiential learning and the hidden curriculum could have the biggest effect on students in that respect [8]. Other suggestions include setting and stating high expectations that students aspire to achieve, treating students respectfully and valuing their opinion by providing them with the opportunity to evaluate their sites and preceptors, and evaluating students’ professional behavior and providing frequent constructive feedback to them [21, 24, 25].

The four different mapping activities employed in this study complemented each other in creating a complete picture asserting that these PPD skills are achieved in the curriculum. Mapping to PEOs and KPC provided confirmation at the highest level of program design since the PEOs are the outcomes guiding the curriculum. However, this specific activity does not provide details for improvements when needed. Mapping of the enacted curriculum by faculty provided insightful guidance for curriculum review and improvement such as transitioning from theory to application of the PPD skills and incorporating them in required courses rather than elective courses. PPD skills should be consistently taught and all students must apply them through the program years. This mapping activity is the most commonly reported one in the literature and serves as a valid method to drive curricular changes [11, 26]. Mapping of the learned curriculum by students triangulated nicely with faculty input as both reported the need to further clarify leadership and management skills, and to systematically provide students with opportunities for application of these skills. Students' perceptions and suggestions were very insightful and illustrated the value of students’ input into curriculum design and revision. Finally, mapping of the assessed curriculum confirmed that not only faculty teach these skills and students are learning them but that the curriculum is evaluating the students for their acquisition of these learning objectives and skills.

This initiative is aligned with the concept of the “eight star pharmacist”, endorsed by the World Health Organization/International Pharmaceutical Federation (WHO/FIP) joint statement on Good Pharmacy Education Practice, which identifies the expected roles and responsibilities of the pharmacists as care-giver, decision-maker, communicator, leader, manager, life-long learner, teacher, and researcher [27, 28]. This initiative has also supported the school’s curriculum committee to further develop self-assessment, professionalism, innovation and leadership throughout the professional degree program. Accordingly, for our program and based on faculty and students’ feedback through the mapping activities, many action items have been adopted that include 1) highlighting the differences between leadership and management to faculty and students through workshops; 2) fostering leadership and management educational outcomes throughout the curriculum; 3) reviewing the syllabi of courses delivering on pharmacy management to include practical experiences for students in management and leadership; 4) developing learning objectives for different student organizations; 5) developing a required course in collaboration with the Outreach and Civic Engagement Unit at the University that would center its objectives on nurturing pharmacy students’ personal and professional skills and provide them with practical experiences that emphasize leadership, innovation and entrepreneurship; and 6) expanding the workshops’ topics offered to faculty and students to include leadership development skills and stress management.

Limitations to this study include the lack of longitudinal follow-up on students’ progress in achievement of PPD-related competencies. As the utilized standardized APPE scoring rubrics provide assessment over a one month block, the study reports on students’ progress over a 5-month period only. It would have been optimal to follow students’ progress on the achievement of the PPD-related competencies through the four professional pharmacy years. In addition, there was potential recall bias from students’ perspectives as they had to rely on memory when reflecting back on examples from the curriculum related to met competencies.

## Conclusion

This study assessed whether the learning objectives of self-assessment, leadership, innovation and entrepreneurship, and professionalism are integrated in a pharmacy program through the mapping of the enacted, learned and assessed curricula. Mapping activities showed the sequential integration of the PPD skills at different depths and breadths in the curriculum. As the current accreditation standards and guidelines are directing pharmacy programs to include and enforce personal and professional development skills, pharmacy programs must develop self-aware and professional leaders who implement innovation and creativity for the continuous improvement of the profession.

## Abbreviations

PPD: Personal and professional development
PEOs: Program educational outcomes
CAPE: Center for the Advancement of Pharmacy Education
ASHP: American Society of Health-System Pharmacists
ACPE: Accreditation Council for Pharmacy Education
LAU: Lebanese American University
SOP: School of Pharmacy
KPC: Key performance criteria
APPEs: Advanced Pharmacy Practice Experiences
P4 students: Fourth professional year pharmacy students
P1 students: First professional year pharmacy students
P2 students: Second professional year pharmacy students
P3 students: Third professional year pharmacy students
IPPEs: Introductory Pharmacy Practice Experiences
OSCE: Objective Structured Clinical Examination
ACCP: American College of Clinical Pharmacy
